# Autoimmune Rheumatic Diseases

**DOI:** 10.1155/2014/952159

**Published:** 2014-08-05

**Authors:** Juan-Manuel Anaya, Yehuda Shoenfeld, Frank Buttgereit, Miguel A. Gonzalez-Gay

**Affiliations:** ^1^Center for Autoimmune Diseases Research (CREA), Universidad del Rosario, Carrera 24 No. 63C-69, 111221 Bogota, Colombia; ^2^Zabludowitcz Center for Autoimmune Diseases, Sheba Medical Center, Tel-Hashomer, Sackler Faculty of Medicine, Tel Aviv University, 52621 Tel-Hashomer, Israel; ^3^Department of Rheumatology and Clinical Immunology, Charité Universitätsmedizin Charitéplatz 1, 10117 Berlin, Germany; ^4^Rheumatology Division, Hospital Universitario Marqués de Valdecilla, IDIVAL, Avenida de Valdecilla, s/n, Cantabria, 39008 Santander, Spain

The term autoimmune rheumatic diseases (ARDs) encompasses a heterogeneous group of conditions characterized by joint involvement along with a wide spectrum of systemic manifestations. The most common ARDs are rheumatoid arthritis (RA) and systemic lupus erythematosus (SLE). Nevertheless, all these conditions share similar pathophysiological mechanisms [1, 2] and a common risk of developing a process of accelerated atherosclerosis [3]. In this regard, in this special issue J. Amaya-Amaya and colleagues discussed the mechanisms associated with the increased risk of cardiovascular disease (CVD) in patients with autoimmune diseases. These authors emphasize the relevance of the CVD in rheumatic conditions and its connection with inflammation and autoimmunity. They also highlight the need of a more aggressive management of these conditions, both of disease activity and classic cardiovascular risk factors. A good example of accelerated atherosclerosis in the setting of an ARD is SLE, in which endothelial dysfunction, an early step in the atherogenesis process, is observed before cardiovascular events can occur. With respect to this, A. Mak and N. Y. Kow performed a comprehensive review of the mechanisms that are involved in endothelial damage. These authors focused on the factors involved in endothelial damage and repair and, therefore, in the development of CVD in patients with SLE. They discussed the relevant role of factors such as type 1 interferon, proinflammatory cytokines, inflammatory cells, immune complexes, costimulatory molecules, neutrophils extracellular traps, lupus-related autoantibodies, oxidative stress, and dyslipidemia that along with the aberrant function of the endothelial progenitor cells lead to endothelial dysfunction and increased susceptibility to develop CVD in patients with SLE. Based on these lines of evidence, the authors' claim is in favor of early intervention at the preclinical stage of atherogenesis in these patients.

Interestingly, damage and activation of vascular endothelial cells are implicated in the pathogenesis of SLE [4]. Angiogenic factors play a significant role in vascular permeability, vascular growth, and inflammatory response observed in SLE. L. Zou and colleagues assessed the serum levels of 3 angiogenic factors in SLE and their clinical significance. These authors disclosed that the levels of PlGF, bFGF, and VEGF are higher in SLE patients with active disease than in those with inactive SLE. Their findings may have a potential interest in the management and development of future therapies for autoimmune diseases.

Besides cardiovascular complications, renal disease and the risk of infection overshadow the outcome of patients with SLE [5, 6]. In this regard, as reported in this special issue by E. Cairoli and colleagues, end-stage renal disease (ESRD) is an important cause of morbidity and mortality in patients with SLE. These authors analyzed the outcome and prognostic factors of renal transplantation in patients with ESRD due to SLE. They assessed 50 renal transplantations that were performed in 40 SLE patients. The most frequent underlying lupus nephropathy that led to ESRD was type IV (72.2%). Graft failure occurred in 30% transplantations and the most common cause of graft failure was chronic allograft nephropathy. The patient survival rate was high. Recurrence of lupus nephritis in renal allograft was only observed in 1 patient. In this study the presence of anti-HCV antibodies and the type of donor source were related to the development of graft failure. According to these results, renal transplantation appears to be a good alternative for renal replacement therapy in patients with SLE.

Since some studies indicate an increased incidence of tuberculosis in patients with SLE [7], a diagnosis of latent tuberculosis infection is of major importance in these patients. M. D. M. Arenas Miras and colleagues report in this special issue a study to compare the tuberculin skin test and the newer T.SPOT.TB test to diagnose latent tuberculosis infection in SLE. Unlike T.SPOT.TB results, the tuberculin skin test results were negatively affected by corticosteroid and immunosuppressive therapy. Because of that, the authors support the use of the T.SPOT.TB test in SLE patients receiving corticosteroids or immunosuppressive drugs.

Patients with RA also have a higher risk for atherosclerosis [8, 9]. E. Gómez-Bañuelos and colleagues from Mexico evaluated the association between membrane expression of CD36 in peripheral blood mononuclear cells (PBMC) and carotid intima-media thickness (cIMT) in patients with RA in order to evaluate the association of membrane expression of CD36 with subclinical atherosclerosis. Other molecules related to cardiovascular risk such as ox-LDL, IL-6, and TNF*α* were also tested. A low membrane expression of CD36 in PBMC from patients with RA presenting with subclinical atherosclerosis and increased serum proinflammatory cytokines was observed.

Proteoglycan-induced arthritis (PGIA) is a widely used model based on the cross-reactivity of injected foreign (usually human) PG and mice self-PG. L. L. W. Ishikawa and colleagues evaluated the arthritogenicity of bovine proteoglycan (PG) and found that it can be used as an alternative antigenic source to PG-induced arthritis for the study of many RA aspects, including the immunopathogenesis of the disease and also the development of new therapies.

Anticitrullinated peptide antibodies (ACPA) are detected in the sera of patients with RA and have a profound role in the diagnosis of the disease [10]. M. L. Díaz-Toscano and colleagues evaluated the performance of using two assays for ACPA: second-generation anticitrullinated cyclic peptides antibodies (anti-CCP2) and antimutated citrullinated vimentin (anti-MCV) antibodies for the diagnosis of RA. Their study suggest that adding the assay of anti-MCV antibodies to the determination of anti-CCP2 increases the sensitivity for detecting seropositive RA, and authors propose the use of both assays in the initial screening of RA in longitudinal studies, including early onset of undifferentiated arthritis.

Since clinical response of biologic agents in RA can be influenced by their pharmacokinetics and immunogenicity, D. Mazilu and colleagues evaluated the concordance between serum drug and antidrug levels as well as the clinical response in RA patients treated with biological agents who experience their first disease exacerbation while being on a stable biologic treatment. Detectable biologic drug levels correlated with a better clinical response in patients experiencing their first RA inadequate response while being on a stable biologic treatment with rituximab, infliximab, and etanercept (ETN).

Interleukin-6 (IL-6), a cytokine that can facilitate autoimmune phenomena, amplify acute inflammation, and promote the evolution into a chronic inflammatory state, has a pivotal role in synovitis, bone erosions, and the systemic features of RA [11]. A comprehensive review on IL-6 and the rationale for blocking this cytokine in RA are also presented in this special issue.

Pharmacogenomics, the study of how genes affect a person's response to drugs, will allow the development of tailored drugs to treat a wide range of health problems, including RA and many others. A. Lima and colleagues report in this special issue the role of methylenetetrahydrofolate reductase (MTHFR) C677T, aminoimidazole carboxamide adenosine ribonucleotide transformylase (ATIC) T675C polymorphisms, and clinicopathological variables in clinical response to methotrexate (MTX) in Portuguese patients with RA. MTHFR 677TT and ATIC 675T carriers were associated with over 4-fold increased risk for nonresponse to MTX. Authors suggest the use of these genotypes combined with clinicopathological data to assist clinicians in personalizing RA treatment.

We hope that readers will enjoy this issue and find accurate data and updated reviews on the most common ARDs.

*Juan-Manuel Anaya* *Yehuda Shoenfeld* *Frank Buttgereit* *Miguel A. Gonzalez-Gay*
